# Population weighted raster maps can communicate findings of social audits: examples from three continents

**DOI:** 10.1186/1472-6963-11-S2-S14

**Published:** 2011-12-21

**Authors:** Steven Mitchell, Anne Cockcroft, Neil Andersson

**Affiliations:** 1 CIETcanada, 1 Stewart Street, Ottawa, Ontario, Canada; 2 CIET Trust Botswana, PO Box 1240, Gaborone, Botswana; 3 Centro de Investigación de Enfermedades Tropicales (CIET), Universidad Autónoma de Guerrero, Acapulco, Mexico

## Abstract

**Background:**

Maps can portray trends, patterns, and spatial differences that might be overlooked in tabular data and are now widely used in health research. Little has been reported about the process of using maps to communicate epidemiological findings.

**Method:**

Population weighted raster maps show colour changes over the study area. Similar to the rasters of barometric pressure in a weather map, data are the health occurrence – a peak on the map represents a higher value of the indicator in question. The population relevance of each sentinel site, as determined in the stratified last stage random sample, combines with geography (inverse-distance weighting) to provide a population-weighted extension of each colour. This transforms the map to show population space rather than simply geographic space.

**Results:**

Maps allowed discussion of strategies to reduce violence against women in a context of political *sensitivity* about quoting summary indicator figures. *Time-series maps* showed planners how experiences of health services had deteriorated despite a reform programme; where in a country HIV risk behaviours were improving; and how knowledge of an economic development programme quickly fell off across a region. *Change maps* highlighted where indicators were improving and where they were deteriorating. Maps of *potential impact of interventions*, based on multivariate modelling, displayed how partial and full implementation of programmes could improve outcomes across a country. *Scale* depends on context. To support local planning, district maps or local government authority maps of health indicators were more useful than national maps; but multinational maps of outcomes were more useful for regional institutions. Mapping was useful to illustrate in which districts enrolment in religious schools – a *rare occurrence* - was more prevalent.

**Conclusions:**

Population weighted raster maps can present social audit findings in an accessible and compelling way, increasing the use of evidence by planners with limited numeracy skills or little time to look at evidence. Maps complement epidemiological analysis, but they are not a substitute. Much less do they substitute for rigorous epidemiological designs, like randomised controlled trials.

## Background

A perennial challenge in social audit, as in most epidemiological research, is to collate and to present evidence in a way that is easily understood by those who most need the information. Visual presentation is a widely accepted strategy for knowledge translation, particularly in settings where people are less numerate or, more often, lack the time needed to comprehend statistics [1]. Maps serve as a visual communication tool in many disciplines, including epidemiology [2]. They can portray trends, patterns, and emphasise spatial differences easily overlooked in tabular data. For example, an overall average indicator of 50% can have regional variation, in an extreme case, from 0% in some communities to 100% in others. Maps allow us to identify hot-spots or outbreaks [3], and to identify the contributing environmental, geographic and social factors [4]. Mapping has been used in evaluation of health services, particularly number, sizes, types, and locations of health services, resource allocation, and how these impact on health and well-being [5,6].

Mapping in health research is not new. John Snow’s London Cholera maps of the mid-1800’s are part of epidemiological folklore, despite debate around the actual role the maps had in the identification of the source of the outbreak [7]. Years before Snow's maps, spot maps illuminated yellow fever epidemics [8]. Modern mapping technologies include remote sensing and geographic information systems (GIS). Hand-held global positioning systems, online mapping applications [9,10], and the emergence of open source GIS [11] have increased the popularity of maps. These tools have illustrated topics such as dengue [9], malaria [12], cancer [13], polio [14], sexual risk behaviours [15], HIV stigma [16] and transactional sex [17]. The term “health geomatics” includes the range of technologies (such as GIS) used to capture, analyse and map health phenomena [18,19].

Much of the literature on mapping in health and health-related research focuses on the analytical approaches available through GIS, such as detection of clustering. Fewer published accounts cover use of the resulting maps to communicate risk and other evidence to planners and community members and how they can inform decision-taking [20,21]. However, some recent examples do exist. For example, Geanuracos described the use of mapping as part of the Connect to Protect project in the United States and Puerto Rico, which helped planners identify areas in need of HIV and STI prevention planning [22]. Jankowski described processes in urban and rural United States where GIS and its products played an integral role in participatory community-based decision taking around water resource planning [23]. Joyce conducted qualitative research among public health decision makers in the United Kingdom and found that they appreciated GIS and mapping, but only as a complement to other sources of information [24]. Driedger and colleagues explored interventions to increase uptake of GIS and mapping for decision making in early childhood learning services in Canada and found only a marginal increase in map use [25,26].

A key concern of social audits [27] is to communicate survey findings to stakeholders at different levels – individual households, local organisations, local governments, national bodies, national governments, and regional groups of governments. In sample communities and local organisations and governments, this generates local evidence-based solutions that aggregate through the representative sample to a coherent policy contribution. At the aggregated level (state or country), the communication seeks to support understanding of the findings and to stimulate action to improve the situation. The evidence-stimulated dialogue on local solutions and actions – socialising evidence for participatory action (SEPA) -- is described elsewhere [28-30]. We present here the approach to producing maps of social audit findings and several ways in which maps have been used as part of SEPA, with examples from different countries and contexts.

## Methods

### Population weighted raster maps

The use of population weighted maps pre-dates modern GIS technologies. For example, in 1929, the Washington Post printed a map of the United States depicting each state with an area relative to its population rather than geographic size [31]. More recently, in the early 1980’s, Taylor produced population weighted heating degree-day maps to help determine potential fuel requirements for Canada [32]. In 1991, Upton demonstrated the use of population cartograms to display election results in the United Kingdom [33]. Andersson and Mitchell described the use of population weighted raster maps, produced using CIETmap software, to display the findings of epidemiological studies [34], illustrating this in a paper exploring the social costs of landmines and the impact of mine risk reduction programmes in Afghanistan [35]. Akin to weather and elevation maps, these population weighted raster maps show colour changes over the study area. Instead of elevations or barometric pressure, data are the epidemiological occurrence – a peak on the map represents a higher value of the indicator in question. The extent of spread of each colour is a function not only of space, as on a weather or elevation map, but also of population. The population relevance of each sentinel site, as determined in the stratified last stage random sample, combines with space (using inverse-distance weighted interpolation) to provide a population-weighted extension of each colour. This transforms the map to show population space instead of simply geographic space. Additional information about the construction of the maps can be found in the accompanying technical annex [see additional file 1 for the technical annex].

The resulting population weighted raster maps can be interpreted much like weather maps, where trends across regions are more informative than values at specific locations. Data are classified into four or five continuous class ranges represented with a legend. This helps to ensure that individual communities are not easily identified, which can be important when communicating sensitive data*.* There is evidence that sensitive information can sometimes be traced back to specific locations, including communities and households, even when these locations are not explicitly included on the original map [36].

A standard colour scheme means that darker areas on the maps represent areas in need of attention or investment. A green colour palette is used for maps that represent “coverage” or “programmes”; a brown colour palette for maps that represent “outcomes”; and a red colour palette for “change” maps. Vector overlays (such as administrative boundaries, roads, or other landmarks) are shown as black or grey lines and are often placed on top of the raster surface to provide visual reference points familiar to the map users. The interpolation itself is independent of the overlay, avoiding a situation where a health outcome changes abruptly at an arbitrary administrative boundary unrelated to the indicator being mapped.

Relying on the CIETmap freeware, we used population weighted raster maps, and extensions of this technique, for communication of evidence from social audits in different contexts and to different target audiences.

## Results

### Presenting sensitive data

Publishing figures about a sensitive topic, such as abuse against women, can detract from efforts to tackle the underlying causes of the problem, as debate centres rather around the accuracy of the summary figures for a country or region. Our large survey in Pakistan in 2003 produced the first nationally representative figures about the levels of different kinds of abuse against women in that country [37]. After the findings were shared with the government ministry that commissioned the research, some officials became concerned that the figures for the levels of different kinds of abuse revealed by the survey were too politically sensitive to be published. Yet they agreed the findings should be shared with government and other stakeholders in each province, in order to help them develop the most effective strategies for tackling the problem. They permitted the use of population weighted maps, including in large scale meetings with invited journalists, provided it did not lead to any “inflammatory headlines” or “negative publicity”.

**Figure 1:**
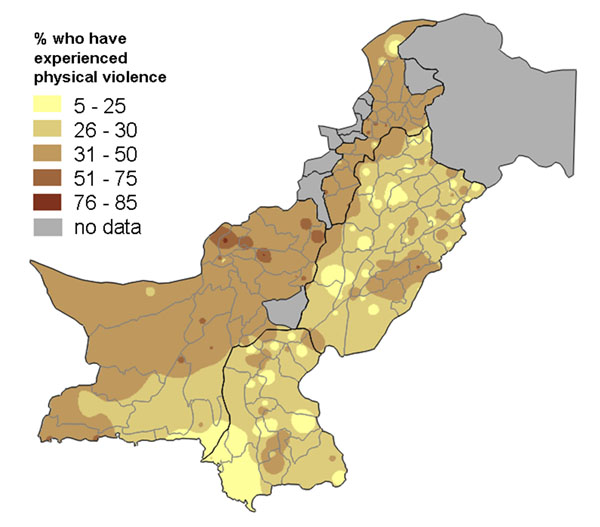
**Proportion of women who have experienced physical violence**. The map shows the variation across Pakistan.

**Figure 2:**
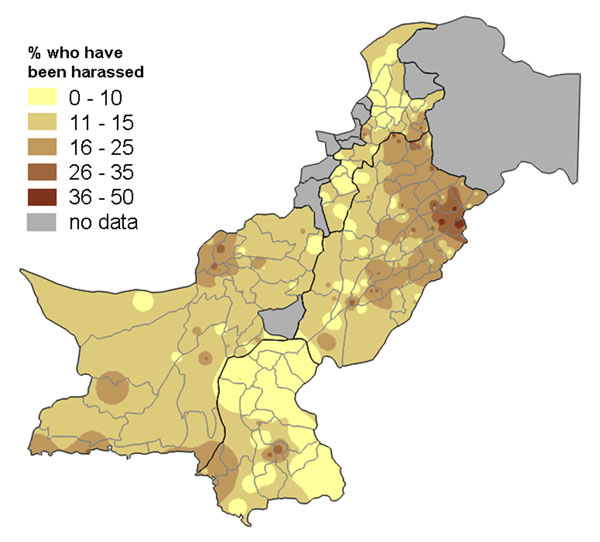
**Proportion of women who have been harassed outside the home**. The map shows the variation across Pakistan.

Population weighted raster maps showed the frequency of abuse against women of different types (for example, Figures 1 and 2). A vector overlay showed the provincial and district boundaries. These maps had a legend that allowed readers or viewers to estimate the approximate levels of abuse in different areas of the country, but the most apparent aspect was the *variation* in the levels of abuse across the country. Figure 1 shows that women in the survey more commonly reported experiencing physical violence in the North West and South West of the country – in this case the pattern of variation coincided quite closely with the provincial boundaries. The grey areas are where no data was collected: the federally administered tribal areas and two districts among the (then) 97 districts in the four provinces. Figure 2 shows there was a different pattern for the experience of harassment outside the home, with this form of abuse being more common in the north of Punjab province, especially around the provincial capital, Lahore. In these areas of the country women are allowed more mobility outside the home, including going to work outside the home. The areas where this form of abuse is less common (much of Khyber Pakhtunkwa, formerly North West Frontier Province, and the interior of Sindh province) are those where women's mobility is typically restricted to within the homestead, unless accompanied by a male family member. Note that the scales on Figures 1 and 2 are different, since harassment outside the home was a less commonly reported form of abuse overall.

We presented these maps of the levels of types of abuse in many meetings across Pakistan, attended by government and civil society stakeholders, as well as the media. In the presentation, for each type of abuse, the map showing the frequency across the country was followed by the results of a multivariate analysis of the variables related to the risk of that type of abuse. The presentation generated considerable interest and discussion, but at no point did anyone ask for the summary national or provincial figures for the levels of different kinds of abuse. Rather the discussion focused on what was responsible for the variation in levels of abuse, and then on how the underlying risk factors could be tackled. The media coverage from the meetings focused on constructive messages about what could help prevent abuse; the journalists did not have summary 'headline' figures to quote. Thus the maps had two advantages: they avoided simplistic, “shock” headlines which may have derailed the whole process of sharing the findings; and they helped to begin the discussion about underlying risk factors and the search for local solutions.

### Presenting changes in time and space

Population weighted raster maps can illustrate temporal changes of programme coverage or impact, giving a visual impression that a table of numbers cannot match. Their advantage over charts (for example bar charts or line plots showing indicators at different time points) is that they show the variation in change across the entity. Maps summarise large amounts of data in an immediately accessible form.

### Time-series maps

A population weighted raster map for a given indicator at the first time point begins a series of maps. Subsequent maps in the series show the same indicator measured at later time points in the same panel of sample communities. Classification values must be fixed across all the maps in the series to ensure that legends are standardized and users can interpret changes on the map accurately. These maps can either be shown side by side, or presented as an animation where one map in the series ‘morphs’ into the next map. Time-series maps can show spatial differences over time that may not be readily apparent from a table. For example, it would be difficult to discern trends in a table showing a panel of 250 sites, with three columns of data showing the proportion of a given indicator across three surveys. Such information displayed on a times-series map can provide an almost instantaneous and visually compelling summary of regional variations in the indicator over time.

Three social audit cycles in Bangladesh in 1999, 2000 and 2003 documented public perceptions, use, and experience of government and other health services during a major health services reform programme [38]. The three surveys visited the same nationally representative sample of 247 communities. Across the three surveys, household opinions of government health services deteriorated, and their use of government services fell. The satisfaction of users of government health services also fell, as did the proportion that reported they received all the prescribed medicines from the health facility.

**Figure 3:**
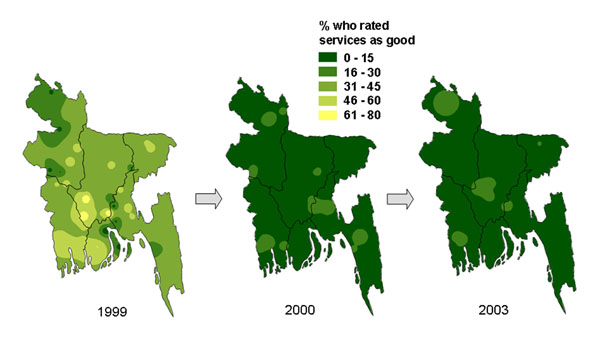
**Proportion of households who rated government health and family planning services as “good”.** The three maps show the variation across Bangladesh between 1999 and 2003.

**Figure 4:**
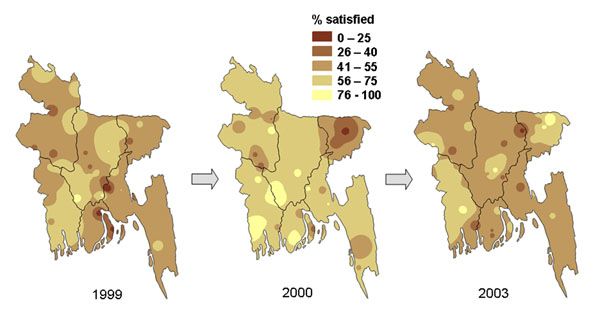
**Proportion of government health service users who were satisfied with the overall service they received**. The three maps show the variation in the change in satisfaction across Bangladesh between 1999 and 2003.

Time series maps helped to illustrate to audiences of government, professional, and civil society stakeholders in Bangladesh how the opinions of the public and the views and experience of government health service users had deteriorated over time, and how this deterioration had happened across the country. In some cases the change was quite uniform across the country, while in other cases it was worse in some parts of the country than in others. Figure 3 shows how public ratings of government health services fell sharply in all parts of the country between 1999 and 2000 and remained poor in 2003. Figure 4 shows how the average figure for the country for overall satisfaction of government health service users, 52% in 1999, 62% in 2000, and 54% in 2003, hides variation across the country at each time point.

**Figure 5:**
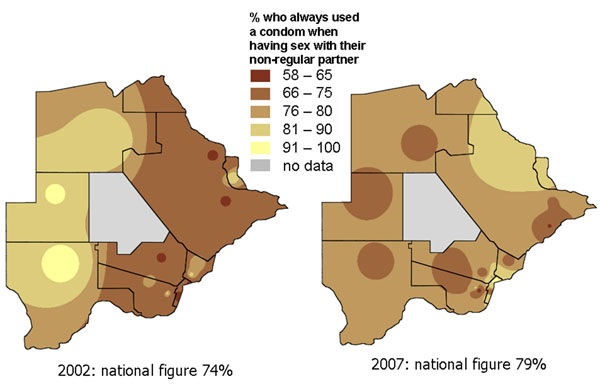
Proportion of adults who always use a condom with a non-regular partner. The two maps show the variation across Botswana in this practice in 2002 and 2007.

**Figure 6:**
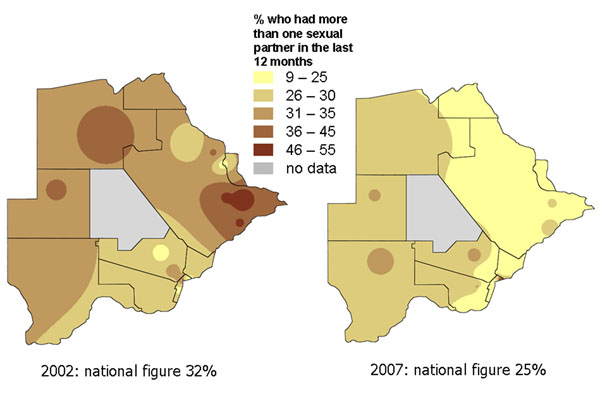
**Proportion of sexually active adults with more than one partner in the last 12 months. **The two maps show the variation across Botswana in this practice in 2002 and 2007.

Two surveys, in 2002 and 2007, collected nationally representative information from households in ten countries in Southern Africa about knowledge, attitudes and practices related to gender based violence and HIV prevention. Time series maps produced for each country helped to share the key findings with national government and non-government bodies responsible for HIV prevention programmes. They illustrated how an average national change in an indicator between 2002 and 2007 could conceal considerable local variation, with some areas improving while others remained static or deteriorated. Figures 5 and 6 show maps of findings shared with programme planners in Botswana [39]. Figure 5 shows the proportion of adults (16-60 years) in Botswana who said they always used a condom when having sex with a non-regular partner. Overall, the proportion always using a condom rose from 74% to 79% between 2002 and 2007. But the maps show that the increase was mainly in the North East, while in the West the proportion using a condom fell over the period. Similarly, Figure 6 shows that the reduction in reported multiple sexual partnerships in Botswana between 2002 and 2007, although it occurred everywhere, was more marked in the North East of the country. Planners from government and NGOs who saw these maps related the pattern to the fact that their programmes for encouraging condom use and partner reduction had been mainly concentrated in the North East, as HIV rates were especially high in that part of the country. They took the findings both as a useful validation of the programmes in the North East, and as an indication that more needed to be done in other parts of the country.

**Figure 7:**
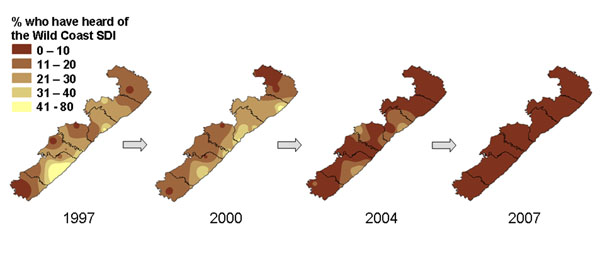
**Proportion of households who had heard of the Wild Coast Spatial Development Initiative. **The four maps show the reduction in knowledge about the SDI across the region between 1997 and 2007.

**Figure 8:**
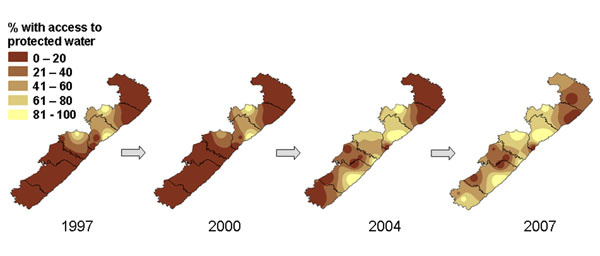
**Proportion of households with access to protected sources of water. **The four maps show the increase in water access across the region between 1997 and 2007.

Figures 7 and 8 show time series of maps of findings from repeated surveys in the Wild Coast area of South Africa [40,41]. Between 1997, 2000, 2004, and 2007 there was a consistent fall in the proportion of respondents who had heard of the spatial development initiative (SDI) in the region, intended to boost the economy and support small enterprises (Figure 7). In 1997, nearly a quarter of respondents overall had heard of the SDI but this had dropped to 10% by 2004, and was almost zero in 2007. The maps show that much of the knowledge in 1997 was centred around the anchor areas – areas where the primary intervention was targeted with the hope that it would spill out to the rest of the region – which appear as the lightest colours on the 1997 map. The time-series maps show that knowledge of the SDI decreased in the region as a whole, including in the anchor areas, and was virtually gone by 2007, after the end of the official SDI programme. Some outcomes did improve over time in the area; Figure 8 is a time series showing how the proportion of households with access to protected sources of water (such as tanks or taps) increased over time. Maps such as these were shared with SDI planners and with communities through focus groups, to guide discussions around why the SDI largely failed and to provide further insight about what was needed from the community perspective.

### Change maps

Change maps are an alternative to time series maps. In this case, the amount of change in an indicator is mapped in a single population weighted raster map, rather than the actual value of the indicator across different time points. The legend is classified into three classes: increase, no change, and decrease. For most outcomes, “no change” can be defined as being within +/- 5%. For rare outcomes +/- 1% would be more appropriate. It is important to decide how “no change” is defined in each case, and make this clear to users of the maps. Change maps give an at-a-glance view of the way an indicator of programme coverage or outcome has changed, not only whether or not it has improved overall but also in which areas the change has been more or less marked. A disadvantage of change maps is that they do not give an indication of the actual level of the indicator; change from a low starting point looks the same as change from a much higher starting point. Therefore, it is often important to use change maps in conjunction with a map or other display of the actual level of the indicator at one of the time points.

**Figure 9:**
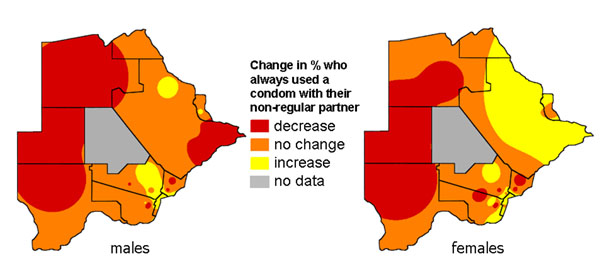
**Change in proportion of adults who always use a condom with a non-regular partner. **The two maps show the change in this practice across Botswana in males and females between 2002 and 2007.

Figure 9 is based on the findings from the surveys in Botswana mentioned above [39]. It emphasizes the spatial heterogeneity of the overall increase in reported condom use in Botswana between 2002 and 2007. In the East and North East there was an increase in condom use, especially among women, while in the West there was a decrease among both men and women. Planners found this display helpful in conjunction with the map in Figure 5 (see above).

**Figure 10:**
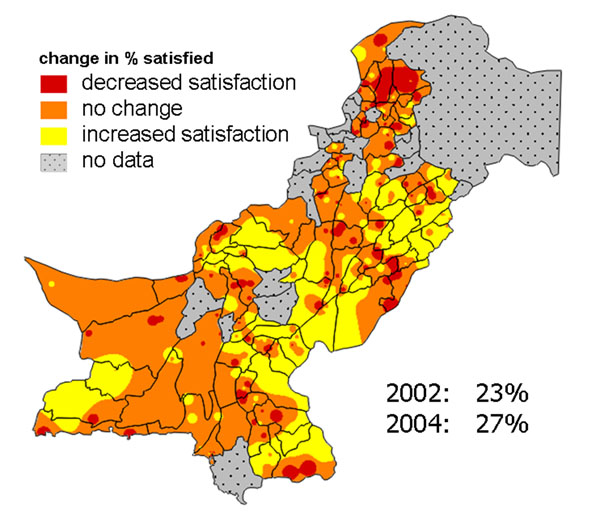
**Change in proportion of households satisfied with government health services**. The map shows the variation across Pakistan in the change (2002-2004) in satisfaction.

**Figure 11:**
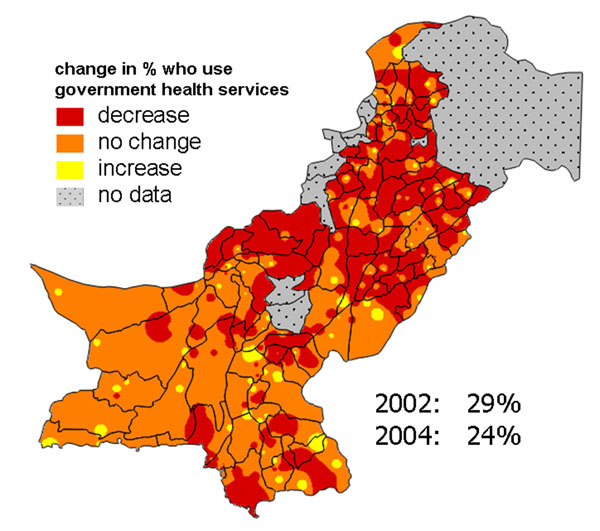
**Change in proportion of households who usually use government health services for medical attention.** The map shows the variation across Pakistan in the change (2002-2004) in use.

In Pakistan, a social audit process examined public perceptions, use, and experience of government health and other services under devolved local government [42]. This included two large nationally representative surveys in 2001-02 and 2004 [43,44]. We used change maps to portray the change in public perceptions and use of services in the first years of devolution to audiences including central and local government and civil society. Figure 10 shows the change in household satisfaction with government health services available to them. Overall, there was a small increase in satisfaction between 2002 and 2004; the change map reveals that in the South East of the country there was an increase in satisfaction in many places, while in the North West there was a decrease in satisfaction. It was important to explain that, even with an overall increase, household satisfaction in 2004 remained low at only 27%. This goes some way to explaining the finding that, between 2002 and 2004, the proportion of households usually using government health facilities for medical attention fell overall, from 29% to 24%. The map in Figure 11 shows this decrease in use of government health services was widespread, especially in the North of the country, while in the remaining areas there was little change.

### Presenting impact scenarios

Presentation of the current level of programme coverage or health outcomes, or even of the way these have changed over time, is insufficient for planners to decide which interventions they should pursue in order to produce the most benefit for the most people – and of course, most cost-effectively. The best way to answer such questions is to conduct a pragmatic randomised cluster controlled trial (RCCT) or a stepped wedge roll-out, randomly allocating areas to receive the new programme first and comparing outcomes in areas with and without the programme after an appropriate time interval [45]. Failing this, it is possible to model effects of different interventions, or combinations of interventions, based on data from cross sectional studies. This modelling of potential population gains is an important aspect of social audit [46]. The challenge is to present the results of this modelling in a way that is accessible to planners, many of whom have limited background in the quantitative sciences. Mapping the potential outcomes of interventions shows how much difference could be made, and in which places the impact might be greatest.

Impact scenario mapping should rely on RCT evidence or on panel data after multivariate analysis. With the final models in place, potential ‘gains’ from an intervention (based on the risk difference and the proportion requiring intervention) are applied to the map, either increasing the value at each site (in the case of a positive phenomenon) or decreasing it (in the case of a negative phenomenon). Gains can either be applied across the map as a whole (for example, a gain of 5% applied to each site) or regional gains can be applied accordingly (for example, if there were different multivariate models for different areas of a country). Each set of intervention impact maps begins with the “current” map of a given indicator (the baseline map with no investment), followed by subsequent maps of different investment scenarios, such as what the map would look like if half of those requiring the intervention had it, and what the map would look like if all of those requiring the intervention had it.

**Figure 12:**
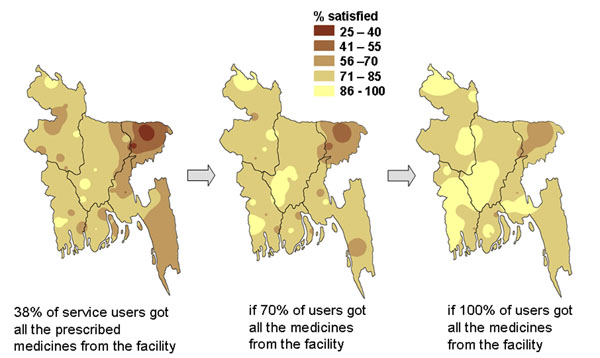
**Potential impact of medicines on proportion of government health services users satisfied with the service. **The maps show the proportion of service users in Bangladesh who would be satisfied under different modelled scenarios.

**Figure 13:**
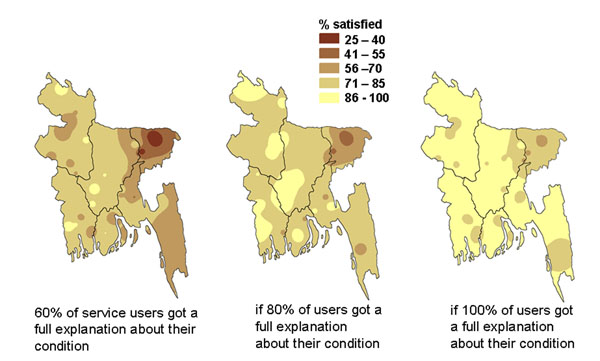
**Potential impact of explanations on proportion of government health services users satisfied with the service. **The maps show the proportion of service users in Bangladesh who would be satisfied under different modelled scenarios.

We used analysis of data from the second of a series of three national household surveys in Bangladesh in 2000 [47] to create maps showing the potential gains in satisfaction of users of government health services if factors associated with lack of satisfaction could be corrected. Two factors strongly related to satisfaction of service users were availability of medicines from the facility and explanation to patients about their condition from health workers. We applied gains at the division level to map the potential impact of changing these two key factors. Figure 12 shows how increasing the proportion of visits in which the service users got all the prescribed medicines from the government health facility could potentially increase the satisfaction of health service users across the country. Figure 13 shows how user satisfaction could potentially increase if more service users got a full explanation about their illness [additional files 2 and 3 show animated versions of Figures 12 and 13]. In the event, the continuing health sector reform programme did not result in increased availability of medicines in the government facilities or improvements in explanations given [38], so it was not possible to examine the “accuracy” of the predictions in practice. After a slight increase between 1999 and 2000, there was no overall increase in satisfaction of service users between 1999 and 2003 (see Figures 4a - 4c).

### Going to scale

For a map to be useful for the intended audience, the scale has to be right. For example, maps showing the variation of an indicator across the whole country are useful for national planners, but less useful for local planners, who need to know how the indicator varies across their area of jurisdiction. This is true whether the map is a population weighted raster map, showing variation of the indicator not demarcated by local authority boundaries, or a vector map displaying the average for each local authority. These maps and the figures underlying them can inform local authorities about how they compare with other local authorities, but they do not help them to plan how to intervene in different parts of their own area of authority.

**Figure 14:**
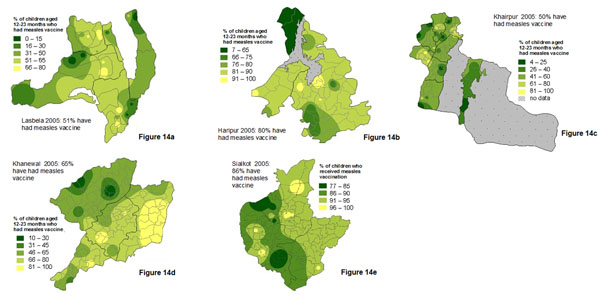
a-e Proportion of children aged 12-23 months who had received measles vaccine, in five districts of Pakistan in 2005.

District *nazims* (elected mayors) attending sessions to discuss the findings of the Pakistan social audit baseline survey [43] noted that the national findings were interesting but they needed locally disaggregated findings, covering issues of particular importance in their district, to support evidence based planning at district level, now more possible under devolution [42]. A social audit process was already under way in a focus district in Balochistan province (Lasbela). Several other districts joined as focus districts and undertook district level social audits, collecting information from enough representative sites in the districts to allow the preparation of district level maps. Figures 14a-14e show the variation in level of childhood measles vaccination across the five focus districts. Note that classification values for the indicator differ between the maps, since the range and average level of immunisation varied considerably between the districts and these maps were intended primarily for internal district use.

**Figure 15:**
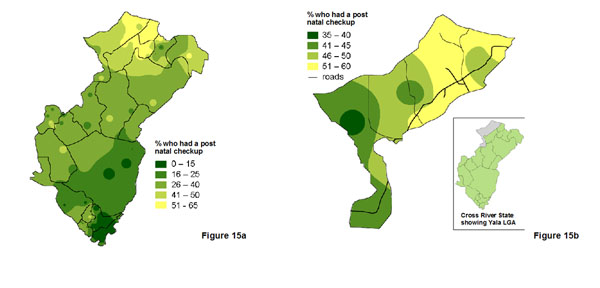
**Proportion of women who had a post-natal check in a health facility in Cross River State, Nigeria, in 2009.** The maps show the variation:a. across the whole state; and b. across Yala LGA.

**Figure 16:**
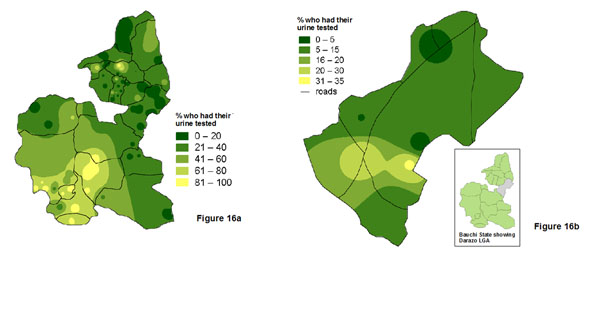
**Proportion of women who had a urine test in a health facility, Bauchi State, Nigeria, in 2009. **The maps show the variation a: across the whole state; and b: across Darazo LGA.

In Nigeria, a social audit focussed on maternal outcomes in two states and in six randomly selected local government authorities (LGA) within these two states [30]. Figure 15a shows the variation in the proportion of new mothers who accessed post-natal care across Cross River state, while Figure 15b shows how the same indicator varies across Yala LGA within the state. The state map fails to capture the whole variation across the LGA, but it is useful to see how the LGA fits into the overall state pattern. Figure 16a shows the variation in the proportion of pregnant women who had a urine test in Bauchi state, and Figure 16b shows how this proportion varies across Darazo LGA within the state. Again, the state map does not reflect all the variation within the LGA, but this is necessary information for planners at LGA level.

At the other end of the spectrum, it can sometimes be useful to prepare maps showing variation across a number of countries, if the intended audience has an overarching role across the whole region. One has to decide whether to prepare vector maps with country as the block unit, or population weighted raster maps, in which the level of an indicator does not necessarily follow national borders.

**Figure 17:**
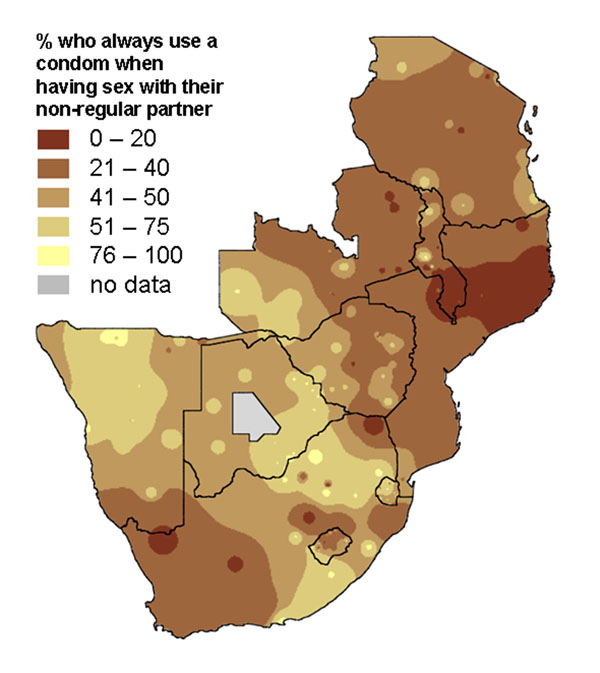
**Proportion of adults who always use a condom with a non-regular partner, 10 SADC countries.** The map shows the variation across the 10 countries in 2007.

A survey in 2007 covered 10 of the 15 countries in the Southern African Development Community (SADC). The survey, about HIV and AIDS related knowledge, attitudes and practice, used the same instrument and data collection methods in all the countries. The research teams presented and discussed the findings in the individual countries. When presenting the findings to SADC bodies with responsibilities across the community, it proved useful to show maps of the key indicators across the geographical area of all 10 countries. These were population weighted raster maps, weighted additionally to allow for the non-proportional sample size in the 10 countries, and with the national boundaries as a vector overlay. Figure 17 shows, for example, the proportion of respondents who reported they always used a condom when having sex with a non-regular partner. There is marked variation across the region.

**Figure 18:**
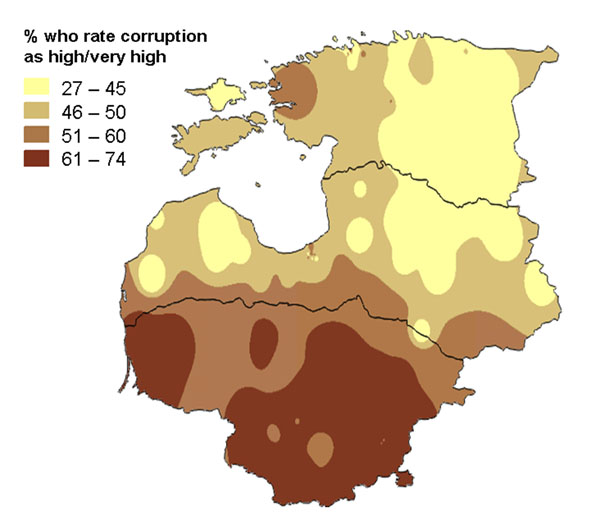
**Proportion of households who rated corruption in government health services as high or very high. **The map shows the variation across Estonia, Latvia and Lithuania in 2002.

**Figure 19:**
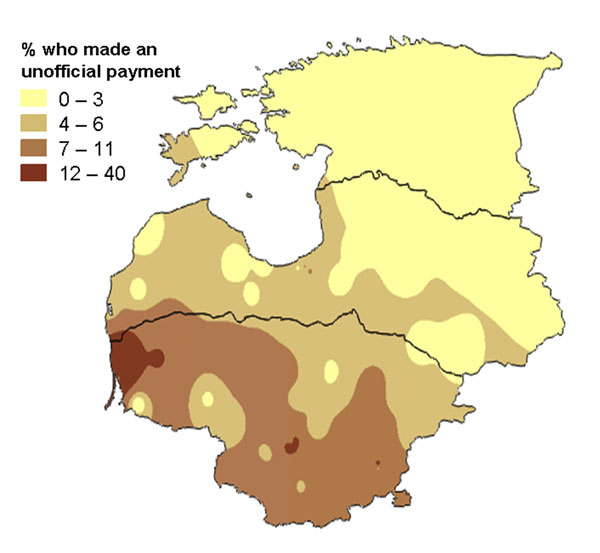
**Proportion of government health service users who made an unofficial payment.** The map shows the variation across Estonia, Latvia and Lithuania in 2002.

The Baltic states of Estonia, Latvia, and Lithuania joined the European Union in 2002. Prior to their entry, they commissioned a survey to examine corruption in several public services, including health services, as part of their joint commitment to tackling corruption. The survey, using the same core instrument in all three countries, covered issues of public perceptions and experience of government health services, including experience of unofficial payments to health workers [48]. As well as discussing the findings in individual countries, the research team also presented the overall findings to joint bodies with representatives from all three countries. For these bodies, maps showing the findings across all three countries were useful. Figures 18 and 19 show two such maps. Note that the indicator spreads across national boundaries but that there are some clear regional patterns. Figure 18 shows that the perception of corruption in health services was higher in the south of the region, with a pocket of high perceived corruption around the capital of Estonia in the north. The personal experience of making unofficial payments (Figure 19) was much lower than the overall perception about corruption, but followed a similar regional pattern.

### Illustrating rare occurrences

Rare events or low prevalence indicators can be difficult to show graphically, especially when their geographic distribution is important. Yet it can be useful to get a picture of where these rare events are located.

**Figure 20:**
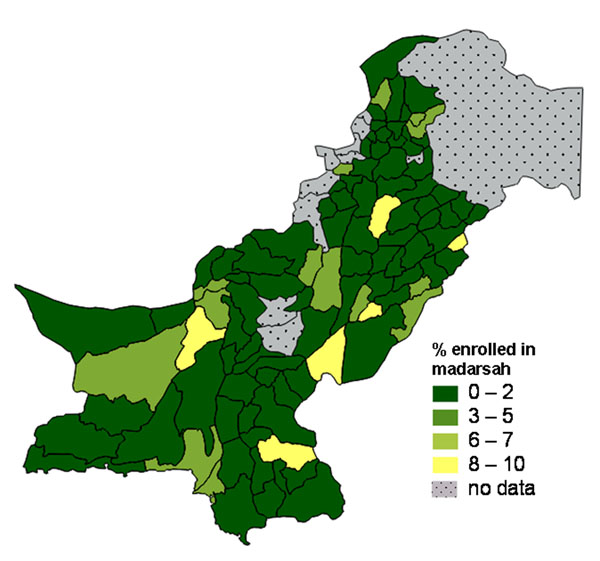
Proportion of all children aged 5-9 years enrolled in a Madarsah (religious school).

**Figure 21:**
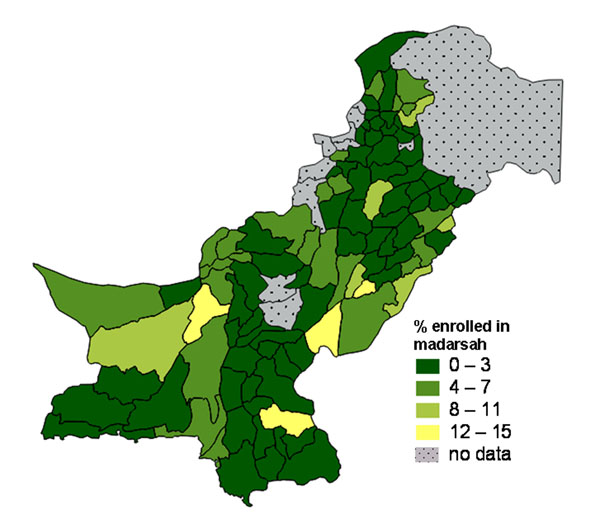
Proportion of school-going children aged 5-9 years enrolled in a Madarsah (religious school).

In Pakistan, a household survey in 2004 collected information about the proportion of children enrolled in *Madaris* (religious schools) [49]. Overall, the proportions enrolled were low but enrolment tended to be concentrated in certain districts. A vector map showed the proportion of all children aged 5-9 years in each district who were enrolled in a *Madarsah* (Figure 20). In most districts less than 2% were enrolled in a *Madarsah*. However, the overall level of enrolment in any type of school was lower in districts in the South West, and this tended to mask the higher proportions of *Madarsah* enrolment in some districts. A more realistic picture of the relative importance of religious education in different districts was provided by a vector map of the proportion of *school-going* children in each district who attended a *Madarsah* (Figure 21). These vector maps proved easier to interpret than raster maps for this low prevalence indicator.

### Limitations and cautions

Maps are a visual aid but in some cases do not help to summarise the findings. For example, in some cases there may be large parts of a country or district that are inaccessible or have little or no population. We typically show areas with “no data collected” shaded in grey. For example, the maps of Pakistan (Figures 1, 2, 10, 11, 20 and 21) have grey areas which are districts or areas where no data was collected; in Botswana (Figures 5, 6, and 9) the grey area represents the central Kalahari reserve with little or no population; in Haripur (Figure 14b), the grey area is a large dam. In these cases, the maps are still useful. But in the case of Khairpur district (Figure 14c), much of the overall district area is mountain or desert with little or no population, and this renders the population weighted map of the district uninformative.

Sometimes, by chance, a random sample of sites across a domain might be concentrated in one part of the domain, or leave out a part. We avoid this whenever possible by including a geographic component as a stratification in the sample selection. Other methods, such as weighted kernel density estimation with an adaptive kernel, can help control for irregular sample distribution [50].

As with any presentation of evidence, it is easy to mislead with maps, intentionally or unintentionally, as described by Monmonier [51]. For example, changing legend categories makes a change appear more impressive than it really is. The same rules of displaying data fairly apply to maps as to any other kind of illustration.

Because one sees a high proportion of outcome Y in one area of a map of a country, and a high proportion of exposure X in the same area of a second map of the country, one cannot conclude that X causes Y. This is a GIS corollary of the well-known ecological fallacy [52,53]. The maps can open a fruitful discussion in this respect. It is not hard to guide the discussion with questions about *individual* relationships with outcome Y, both in areas where the outcome is common and in areas where the outcome is less common, and that the exposure X might not be the real cause of outcome Y at all. This can help to guide the formal epidemiological analysis of potential confounders.

## Conclusions

Population weighted raster maps can present social audit findings in an accessible and compelling way. People with limited numeracy skills (or very little time to look at evidence) can readily appreciate spatial variation and changes over time in maps. Maps are powerful and persuasive and are a useful complement to epidemiological analysis, but they are not a substitute for in depth analysis. Much less do they substitute for rigorous epidemiological designs, like RCTs. It is important to match the type and scope of the map to the intended audience. Our examples illustrate the range of maps that can be produced and their usefulness and limitations.

## List of abbreviations used

GIS: Geographic Information System; LGA: Local Government Authority; NGO: Non Government Organisation; RCCT: Randomised Cluster Controlled Trial; RCT: Randomised Controlled Trial; SADC: Southern African Development Community; SDI: Spatial Development Initiative; SEPA: Socialising Evidence for Participatory Action

## Competing interests

The authors declare they have no competing interests.

## Authors' contributions

SM assisted with the development of CIETmap software, produced the maps in the figures and contributed to drafting the paper; AC undertook many of the surveys whose results are illustrated in the maps and contributed to drafting the paper; NA designed and developed the CIETmap software, supported the surveys whose results are mapped, and contributed to drafting the paper.

## Supplementary Material

Additional file 1 **Technical annex - population weighted and inverse distance weighted spatial interpolation for epidemiological mapping**. Click here for file

Additional file 2 **Animation of Figure **12, **showing potential benefits on patient satisfaction of drug availability in Bangladesh** The file allows readers to see animations of the data displayed in Figure 12. Click here for file

Additional file 3 **Animation of Figure**13, **showing potential benefits on patient satisfaction of health provider explanations in Bangladesh** The file allows readers to see animations of the data displayed in Figure 13. Click here for file
