# Supplementary material for: Population weighted raster maps can communicate findings of social audits: examples from three continents
Source: BMC Health Serv Res. 2011 Dec 21;11(Suppl 2):S14. doi: 10.1186/1472-6963-11-S2-S14 (PMC3332558; doi:10.1186/1472-6963-11-S2-S14)
Supplement: Additional file 1 — Technical annex - population weighted and inverse distance weighted spatial interpolation for epidemiological mapping. [file 1472-6963-11-S2-S14-S1.pdf]

Technical annex

Population weighted and inverse distance weighted spatial interpolation for epidemiological mapping

Steven Mitchell[1], Neil Andersson[2] and Mario Beauchamp[1]

[1]CIETcanada, 1 Stewart Street, Ottawa, Ontario, Canada

[2]Centro de Investigación de Enfermedades Tropicales, Universidad Autónoma de Guerrero, Acapulco, Mexico

Email addresses:

SM: smitchell@ciet.org

NA: andersson@ciet.org

MB: mbeauchamp@ciet.org

This is the technical annex to: Mitchell S, Cockcroft A, Andersson N. Population weighted raster maps can communicate findings of social audits: examples from three continents. *BMC Health Services Research* 2011, 11(Suppl 2): S14.

The concept and interpretation of population weighted raster maps

Spatial interpolation is a way of generating a surface of values with data from sampled locations. Widely used methods of spatial interpolation include inverse distance weighting (IDW) and kriging [1]. There is a strong literature comparing the accuracy and appropriateness of different approaches [3, 4, 5, 6].

CIET's approach to evidence-based planning collects household and individual data from a panel of sample communities that together represent the target population [2]. We weight each selected community to keep the sample characteristics proportional to the population. In order to interpolate data from these sample communities on raster maps, we use IDW adjusted to take into account the population weights. The result is a population weighted estimate with an indicative spatial spread (see Figure 1).

Figure 1: % of households with access to protected sources of water (Wild Coast SDI 2007)

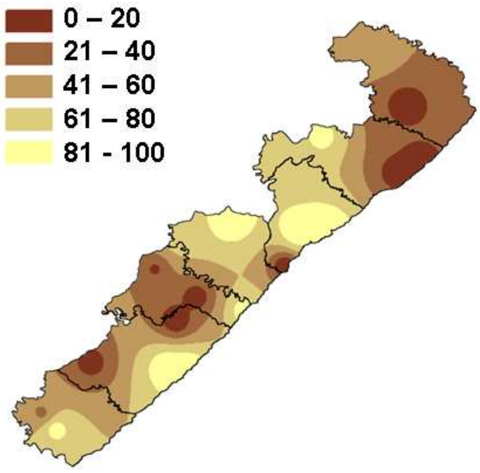

We do not intend these maps to be interpreted literally, with all the people living under each contour line experiencing the conditions described by that contour line – as they might, for example, if it was a weather map. We intend the map to be viewed as a whole, resting on the fact that the site selection represents the domain, in Figure 1 the Wild Coast of South Africa. Our link between spacial extension and population conveys a sense (i) of the broad location of hotspots and cold-spots across the domain, and (ii) how many people are in each category. In this example, if 10% of the map has the lightest colour, reflecting 81-100% with access to protected water, the interpretation is that only 10% of the communities report this level of access.

We use population weighted raster maps for communication of evidence-based research in different contexts and to different target audiences such as individual households, local organisations, local governments, national bodies, national governments, and regional groups of governments. Such audiences are often not familiar with numeric-based tools, and as a result tables or reports of findings have little use. Maps provide a visual display of data that is easily understood by people familiar with the domains portrayed.

Calculation of population weights

CIET social audits almost all use last stage random (LSR) stratification to select sample communities. Criteria for stratification changes with each setting and may include urban/rural status, remoteness, and administrative division (to ensure data is collected from each province or state within a country). We allocate the number of sites to each stratum proportional to population, and then choose sites randomly from within each stratum.

For example, to select 100 sites from a population that is 20% urban and 80% rural we would randomly select 20 from a list of all urban sites and, separately, randomly select 80 from a list of all rural sites. The stratification ensures the sample is broadly proportional to the population with respect to urban and rural status. However, it does not guarantee proportionality with respect to other characteristics, like density of population or response rates. Some of these may be balanced by the LSR sample, but there is no guarantee this will be the case with a limited number of sites. We use population weighting to re-establish proportionality for the main variables.

Table 1: Transparency table for the calculation of population weights

	% of population	% of sample	population weight
Urban sites	20%	40%	0.50
Rural sites	80%	60%	1.34

Table 1 shows a transparency table used to estimate population weights. In this example, the sample was 40% urban and 60% rural. Urban households were over-represented in the sample and we need to reduce the influence of this on derived indicators. If the study was looking at access to services, for example, this urban bias could be relevant to the interpretation of the result. In order to calculate the weights we divide the *% of population* by the *% of sample*. We then use the resulting population weights to create "weighted averages" of the indicators in question. In this scenario, the population weights will correctly reduce the overall effect of urban sites on the final result, eliminating the urban bias on the result.

Population weighted mapping

The concepts behind IDW are easy to explain to stakeholders relative to other interpolation techniques, such as kriging. Non-geographers have an intuitive sense of Tobler's first law of geography that "nearer things are more closely related than further things" [7]. IDW builds on this. The formulas for IDW have been published elsewhere (for example, Babak & Deutsch 2009 [8]). The weights are calculated as a function of inverse distance, meaning that closer locations with have "more weight" on an unknown interpolated value than further locations. The degree of this is controlled by the distance-weight exponent, where a value of 2 is commonly used (ie: $1/d^2$) [8].

Figure 2: Standard inverse distance weighted interpolation between two sites with a distance weight exponent of 2

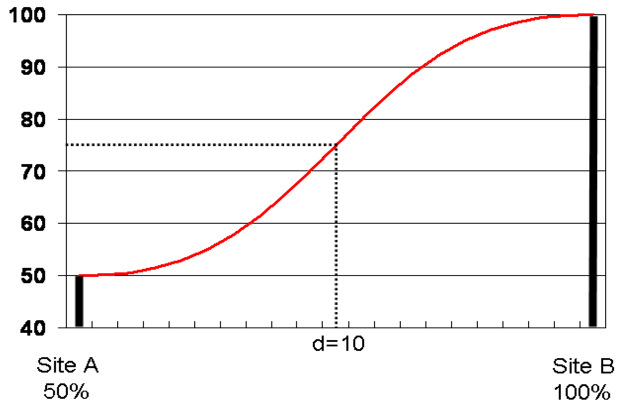

Figure 2 depicts a cross-sectional view of an inverse distance weighted interpolation between two points, with a distance weight exponent of 2. In this example, Site A has a value of 50%, and Site B has a value of 100% for an indicator. This is analogous to elevations, but where the phenomena is "height" of epidemiological data (such as % exposed to a given intervention).

Regardless of the distance weight exponent used, the value at the mid-way point – in this case 10 units - will be the average between the two values. If no distance weight exponent was used, simple linear interpolation would result, and the mid-way interpolated value would remain unchanged (in this case, 75%).

Our implementation of IDW uses global interpolation, so that every sample site in the study area is used in the calculation rather than a local, or neighbourhood-based interpolation. IDW preserves the values at the sampled locations and no interpolated value exceeds the maximum or minimum recorded value, which is appropriate for evidence-based research based on sampling. We adjusted the IDW formula to account for population weighting in addition to distance weighting. This accounts for the possible bias introduced by sample sites that are either over- or under-represented based on their population characteristics.

Figure 3: Population weighted inverse distance weighting formula

$$PWIDW = \frac{\sum_{i=1}^{n} v_i \, w_i \, (1/d^x_i)}{\sum^{n} w_i \, (1/d^x_i)}$$

In our early versions of CIETmap, formulas were closed to users, and we had no way to incorporate the population weighting at the time of interpolation. As a result, adjustments were made post-interpolation[9]). Open-source GIS applications [10, 11] now make it possible to incorporate population weighted adjustment at the time of interpolation using the formula shown in Figure 3 (where v represents the value at each sampled location; w represents the population

weight assigned to each sample location; d represents the distance from each unknown point to each sampled location; and x represents the distance weight exponent). This adjustment determines how much influence each community has upon the final map based on its population weight, much as it does for a ‘weighted average’ calculation.

Figure 4: Comparison between standard IDW, and population weighted IDW

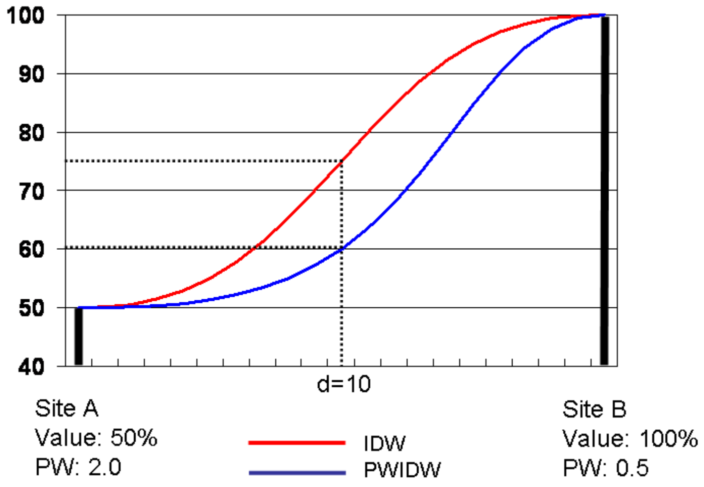

Figure 4 depicts the same cross-sectional view of an interpolation between two sites, but highlights the difference between IDW without population weighting, and IDW that takes the population weighting into account (PWIDW). In this example, Site A is under-represented in the sample, and has been given a calculated population weight of 2.0. Conversely, Site B is over-represented in the sample and has been given a calculated population weight of 0.5. The result of the population weighted interpolation can be demonstrated by the derived value at the mid-way point, which is 75% in the unweighted calculation and 60% in the weighted calculation. It is important to note that the distance weight exponent is the same in both cases. Therefore the difference is a result of the population weighting.

Figure 5 depicts examples of standard IDW and population weighted IDW interpolated rasters, with a contour gradient divided into five equal-interval classes of 10%. The proportion of the map in the darker colours is accurately increased in the population weighted map. Such a transformation allows a planner to think of the map in terms of population space, rather than simply geographic space.

Figure 5: Depiction of IDW and PWIDW interpolation between two points, classified into five equal-interval categories

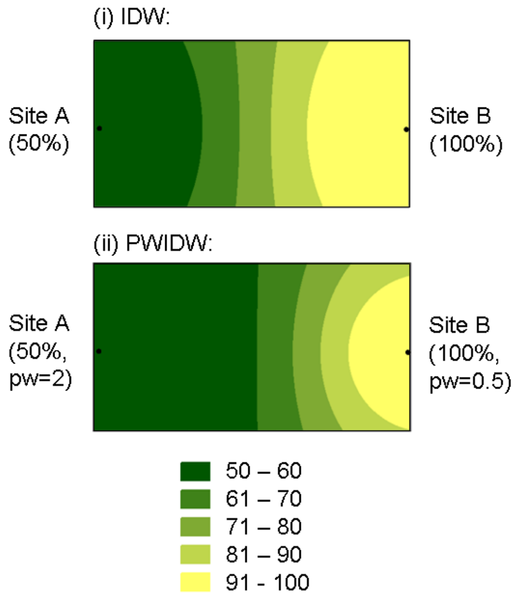

Competing interests

The authors declare they have no competing interests.

Authors' contributions

SM assisted with the development of CIETmap software and PWIDW formula, produced and contributed to drafting the paper; NA designed and developed the CIETmap software and PWIDW formula. MB programs the open source CIETmap software which incorporates the PWIDW formula.

References:

1. Li J, Heap AD: **A review of comparative studies of spatial interpolation methods in environmental sciences: Performance and impact factors**. *Ecological Informatics* 2011 (in press), doi:10.1016/j.ecoinf.2010.12.003.

2. Andersson N: **Evidence-based planning: the philosophy and methods of sentinel community surveillance.** Washington D.C.: World Bank Economic Development Institute; 1996.

3. Wong D, Yuan L, Perlin SA : **Comparison of spatial interpolation methods for the estimation of air quality data.** *Journal of Exposure Analysis and Environmental Epidemiology* 2004, 14(5): 404-415.

4. Mueller TG, Pusuluri NB, Mathias KK, Cornelius PL, Barnhisel RI, Shearer SA: **Map quality for ordinary kriging and inverse distance weighted interpolation.** *Soil Science Society of America Journal* 2004, 68:2042–2047.

5. Zimmerman D, Pavlik C, Ruggles A, Armstrong MP: **An experimental comparison of ordinary and universal kriging and inverse distance weighting.** *Mathematical Geology* 1999, 31(4): 375-390.

6. Weber D, Englund E: **Evaluation and comparison of spatial interpolators.** *Mathematical Geology* 1992, 24(4): 381-391.

7. Sui DZ: **Tobler's First Law of Geography: A Big Idea for a Small World?** *Annals of the Association of American Geographers* 2004, 94(2): 269–277.

8. Babak O, Deutsch C: **Statistical approach to inverse distance interpolation**. *Stochastic Environmental Research and Risk Assessment* 2009, 23:543–553.

9. Andersson N, Mitchell S: **Epidemiological geomatics in evaluation of mine risk education in Afghanistan: introducing population weighted raster maps**. *International Journal of Health Geographics* 2006, **5**(1): doi:10.1186/1476-072X-5-1.

10. Steniger S, Bocher E: **An overview on current free and open source desktop GIS developments**. *International Journal of Geographical Information Science* 2009, **23**(10): 1345-1370.

11. Andersson N, Mitchell S: **CIETmap: Free GIS and epidemiology software from the CIET group, helping to build the community voice into planning**. *Presentation*. World Congress of Epidemiology; August 19, 2002; Montreal, Canada.
